# Occupational injuries among children and adolescents in Cusco Province: a cross-sectional study

**DOI:** 10.1186/1471-2458-14-766

**Published:** 2014-07-30

**Authors:** Cornelia Schlick, Manuela Joachin, Leonardo Briceño, Daniel Moraga, Katja Radon

**Affiliations:** Unit for Occupational and Environmental Epidemiology & Net Teaching, CIHLMU @ Institute and Outpatient Clinic for Occupational, Social and Environmental Medicine, University Hospital Munich (LMU), Ziemssenstraße 1, 80336 Munich, Germany; Clinic and Policlinic for Orthopedics, Physical Medicine and Rehabilitation, University Hospital Munich, Marchioninistrasse 15, 81377 Munich, Germany; Centro Medico Espinar SRL, RPC 968211042 Cuzco, Perú; Centro de Asesoría y Consultoría en Salud Ocupacional, Universidad del Rosario, Calle 12C No. 6-25, Bogotá, Colombia; Facultad de Medicina Universidad Diego Portales & Asesor OFECS, Universidad Iberoamericana de Ciencias y Tecnología, Santiago, Chile

**Keywords:** Child labor, Cusco, Occupational injuries, Perú

## Abstract

**Background:**

Although the number of child laborers in Latin America is generally high, data on occupational hazards and injuries is insufficient. The objective of this study was therefore to determine the lifetime prevalence of and risk factors for occupational injuries among working students (10–17 years old) in Cusco Province.

**Methods:**

A cross-sectional study was conducted at five public night schools. 375 students (response 91.5%) completed an interview-based questionnaire on socio-demographics, work-related factors, and lifetime prevalence of occupational injuries. Multiple logistic regression analyses were performed to estimate risk factors for different types and causes of occupational injuries.

**Results:**

Falls (11%), car accidents (9%) and physical violence (3%) were common causes of injuries in this population. Severe injuries (fractures, luxation or amputations) were reported by 3% of the population. A high daily income (≥20 PEN, ~15 USD) was a statistically significant predictor for injuries caused by falls [OR 2.8; 95% CI 1.2-6.5] and physical violence at work [12.1; 1.3-115.9] whereas children born in Cusco and those working in the service sector were at higher risk of injuries caused by car accidents [3.7; 1.5-9.3 and 4.2; 1.2-15.3].

**Conclusions:**

Occupational accidents among child workers attending public night schools are common in Cusco with a lifetime prevalence of 3% for severe injuries. High income seems to convince child laborers to accept poor working conditions.

## Background

There are some 215 million child laborers in the world of whom more than 14 million are working in Latin America and the Caribbean [1]. Child labor continues to be a health hazard, affecting the mental, physical and emotional wellbeing of young workers [2, 3]. Minimum age for different types of employment is defined by international standards: age 13 for light work, age 15 for ordinary work and age 18 for hazardous work [1]. While the number of working children seems to be decreasing, particularly among girls and young children, there is an alarming trend towards a growing number of adolescents working under hazardous conditions [1]. Hazardous work is defined as “any activity or occupation that (…) has adverse effects on the child’s safety, health and moral development”, including conditions such as working in harmful environments, handling dangerous machinery and heavy loads, being forced to live and work separated from home or working long hours.^1^ Hazardous child labor can be observed over a wide range of occupational sectors [4].

In Perú, there are approximately 2 Million working children and adolescents, nearly 28% of the 6–17 years old Perúvian population [5]. About 77% of them are working while attending school. Most of these children are employed in the agriculture (~37%), retail (~17%), domestic service (~9%) and manufacturing (~9%) sectors [5]. Geographically, the prevalence of child and adolescents labor is highest in the high mountains of the Andes region and decreases continuously towards the coastal region [5]. Data on occupational health of working children in the metropolitan area of Lima are available from one transnational study carried out in Lima, Bogotá, Quito and Sao Paulo. In this survey it was shown that about 40% of the working children had suffered occupational injuries. The most prominent risk factors for injuries were working long hours and performing on the street [6]. Results from Lima, where a comparative small percentage of children are working (12.5%), do not necessarily apply for other Perúvian - especially rural - regions. In rural areas, children are predominantly working in the agriculture sector, whereas in urban areas they are mainly engaged in construction and commerce [7]. In addition, tourism is a growing field of occupation within some parts of Perú but occupational characteristics of children working in touristic areas, have not been studied yet.

In the region of Cusco, 42% of children between 6 and 17 years are estimated to be involved in occupational activities, ranking the region sixth out of 25 within the whole country [5]. Within this region, especially the province of Cusco has become a popular touristic destination with increasing urbanization and raising job opportunities, including for children and adolescents. Many of the jobs for children are offered in the service sector, including working in hotels and private accommodation facilities. Other children offer their services such as shoe polishing or cleaning cars on the street. These activities can be associated with hazards like long working hours and night shifts, noise and high temperatures as well as verbal and physical violence and traffic accidents [4]. We therefore chose Cusco province to study the lifetime prevalence of work-related injuries and to identify risk factors for the occurrence of occupational injuries among children and adolescents attending public night schools.

## Methods

### Participants and procedure

A cross-sectional survey was conducted at all public night schools in Cusco Province. Cusco Province (capital city: Cusco) is divided into eight districts. During the time of the study, there were five public night schools in Cusco Province, located in the districts of Cusco, Wanchaq, San Sebastian, San Jeronimo and Santiago. In 2008, the population size of Cusco Province was about 370,000 inhabitants of which 36% were younger than 18 years [8].

Data were collected between August and December 2010. A total of 410 students between 10 and 17 years old were cluster sampled from the primary and secondary school level. An interview-based questionnaire was administered by the project coordinator (MJ) who visited the students at school. Participation in the study was voluntary (response 91.5%). Informed consent was provided by each student as well as the school directors. The study protocol was approved by 1) the Ethics Committee of the University Hospital Munich (LMU) and 2) the Ministerio de la Mujer y Desarrollo Social (Ministry of Women and Social Development) in Perú.

### Survey instrument

The questionnaire was developed and tested by the Universidad Del Rosario in Bogotá, Colombia. To date it has been used in various cities and settings in South America [6]. Considering demographic and cultural differences between Cusco and other Latin American cities, some questionnaire items on school grade, child’s occupation, and income were adapted to the local situation.

The modified version of the questionnaire consisted of 23 closed items addressing socio-demographics (gender, age, minority status, school grade, residential status and health care coverage), the child’s occupation and working conditions as well as the occurrence and nature of occupational injuries.

For the information on the child’s occupation, 12 response options were provided: *selling products (such as clothes, candy, handcrafts, etc.)*, *cleaning or guarding cars*, *begging*, *recycling*, *performing*, *stealing*, *drug dealing*, *other. Domestic work*, *shoe polishing* and *transporting goods* were added as a modification to the questionnaire. None of the children reported being involved in *begging*, *performing*, *stealing* and *drug dealing*. Most common “other” jobs were *working as bus attendant*, *carpentering* and *construction work*. For the analyses, occupations were grouped into four job sectors: retail (*selling products*), service (*cleaning or guarding cars, recycling, domestic work including accommodation facilities, shoe polishing, transporting goods*), carpentering and construction. Other questionnaire items addressed working conditions included workdays per week, income per day and the child’s work environment.

For the information on occupational injuries, children were asked whether or not they ever had suffered from 1) burns, 2) cuts/lacerations, 3) scratches/abrasions, 4) amputations, 5) car accidents or 6) other injuries while working. In the case of “other”, children were asked to specify the nature and severity of the injury. These included pricks, injuries caused by falls and physical violence, fractures and luxation. Occupational injuries were then classified according to either the outcome or the cause of injuries: 1) mild injuries (cuts, abrasions, pricks, and mild burns), 2) severe injuries (fractures, luxation and amputations), 3) injuries caused by falls, 4) injuries caused by car accidents and 5) injuries caused by physical violence at work.

### Data analyses

All data were double entered in an Excel database and analyzed using the SPSS statistical software package 19 (Chicago, IL).

The absolute and relative frequencies of socio-demographic factors, occupational sectors, working conditions and occupational injuries were calculated.

To identify potential risk factors for 1) injuries caused by falls, 2) injuries caused by car accidents, 3) injuries caused by physical violence at work and 4) severe injuries (fractures, luxation and amputations), bivariate associations between socio-demographics, occupational factors and each of the four groups of occupational injuries were computed using the Chi^2^-test. Associations with a  ≤ 0.1 were considered as potential risk factors and selected for the final multiple logistic regression models.

## Results

### Descriptive characteristics

There were 375 children and adolescents in the study sample. At the time of the study, all of them were working while attending night schools in Cusco (Table 1). Mean age was 14.9 years (standard deviation 1.6 years), ranging from 10 to 17 years. The majority of the students had moved into the city (89%), most of them escaping from poverty (46%) or seeking better opportunities (31%).

**Table 1:** **Sociodemographics of 375 child laborers in Cusco Province, total and stratified for gender and age**

Category	n _missing_	n (%)	n (%)	n (%)	n (%)	n (%)
			male	Female	9–14 years	15–17 years
**Age (years)**	0					
10–14		143 (38.1)	81 (37.5)	60 (38.7)		
15–17		232 (61.9)	135 (62.5)	95 (61.3)		
**Gender:**	4					
**Male**		216 (58.2)			81 (57.4)	135 (58.7)
**Female**		155 (41.8)			60 (42.6)	95 (41.3)
**Race**	5					
Blanco		0 (0.0)	0 (0.0)	0 (0.0)	0 (0.0)	0 (0.0)
Afroamericano		0 (0.0)	0 (0.0)	0 (0.0)	0 (0.0)	0 (0.0)
Mestizo		218 (58.9)	140 (65.7)	75 (49.0)	82 (65.7)	136 (59.1)
Indigena		152 (41.1)	73 (34.3)	78 (51.0)	58 (34.3)	94 (40.9)
**Schooling**	5					
Primary school		115 (31.1)	64 (29.9)	49 (32.2)	63 (45.0)	52 (22.6)
Secondary school		255 (68.9)	150 (70.1)	103 (67.8)	77 (55.0)	178 (77.4)
**Health care coverage**	8	25 (6.8)	12 (5.7)	13 (8.6)	9 (6.5)	16 (5.7)
**Born and raised in Cusco**	6	40 (10.8)	26 (12.2)	13 (8.6)	11 (7.9)	29 (12.7)
**Reason for moving to Cusco**	15					
Violence		11 (3.1)	7 (3.8)	4 (3.0)	7 (5.6)	4 (1.1)
Better opportunities		111 (30.8)	66 (36.1)	45 (33.3)	41 (33.0)	70 (19.4)
Poverty		164 (45.6)	95 (51.9)	68 (50.4)	60 (48.4)	104 (28.9)
Education		14 (3.9)	7 (3.8)	7 (5.2)	8 (6.5)	6 (1.7)
Other		20 (5.6)	8 (4.4)	11 (8.1)	8 (6.5)	12 (3.3)
**Living status: alone**	9	89 (23.7)	47 (22.1)	40 (26.7)	12 (8.6)	77 (33.9)
**Income (per day)**	27					
0–9 PEN		153 (44.0)	69 (33.7)	82 (59.0)	82 (61.7)	71 (33.0)
10–19 PEN		127 (36.5)	78 (38.0)	47 (33.8)	41 (30.8)	86 (40.0)
≥ 20 PEN		68 (19.5)	58 (28.3)	10 (7.2)	10 (7.5)	58 (27.0)

The occupational sectors and working conditions are presented in Table 2. Almost 60% of the students worked in the service sector, 56% of whom were doing domestic work. Only 5% were involved in construction. The mean number of workdays per week was 6.3 days (range 1–7 days). Mean income per day was 11.3 PEN (~4.3 USD) (standard deviation 7.8 PEN; ~2.9 USD). Assuming 6 workdays per week, this adds up to a monthly income of about 270 PEN (~103 USD) compared to a minimum monthly wage of 750 PEN (~286 USD) in Perú. Three children reported to have no income at all. One of them was helping the family with whom he was living as a carpenter. The other two were helping their family in the service sector. Most of the students reported to work under ergonomically poor conditions, such as repetitive work and handling heavy devices (82%) and about two-thirds stated to be exposed to physical pollutants (68%) or some form of psychosocial strain (67%).

**Table 2:** **Occupational sectors and working conditions of 375 child laborers in Cusco Province, total and stratified for gender and age**

Category	n _missing_	n (%)	n (%)	n (%)	n (%)	n (%)
			male	female	9–14 years	15–17 years
**Occupational sector**	1					
Retail		89 (23.8)	51 (23.7)	38 (24.5)	38 (26.6)	51 (22.1)
Manufacturing		41 (11.0)	34 (15.8)	6 (3.9)	15 (10.5)	26 (11.3)
Service		224 (59.9)	110 (51.2)	111 (71.6)	84 (58.7)	140 (60.6)
Construction		20 (5.3)	20 (9.3)	0 (0.0)	6 (4.2)	14 (6.0)
**Any exposure to physical pollutants**	7	**251 (68.2)**	**183 (85.9)**	**64 (42.4)**	**96 (69.1)**	**155 (68.0)**
Of these:						
Noise exposure	11	97 (26.6)	83 (39.2)	13 (8.7)	33 (24.1)	64 (28.2)
Exposure to high or	7	220 (59.8)	165 (77.8)	52 (34.4)	89 (65.0)	131 (57.7)
low temperature						
Dust/smoke exposure	10	230 (63.0)	173 (81.6)	54 (25.5)	90 (65.2)	140 (61.7)
**Any ergonomic factor**	6	**302 (82.5)**	**185 (86.9)**	**113 (74.3)**	**111 (79.3)**	**191 (83.4)**
Of these:						
Heavy physical work	6	125 (33.9)	93 (43.7)	31 (20.4)	41 (29.3)	84 (36.7)
Repetitive work	9	295 (80.6)	182 (85.4)	109 (70.3)	106 (76.3)	189 (83.3)
**Any exposure to psychosocial strain**	7	**245 (66.6)**	**139 (65.3)**	**102 (67.5)**	**87 (62.1)**	**158 (69.3)**
Of these:						
Low salary	12	104 (28.7)	53 (25.2)	49 (32.9)	40 (29.2)	64 (28.3)
Long workdays	8	188 (51.2)	116 (54.7)	70 (46.4)	63 (45.3)	125 (54.8)
Violence	7	73 (19.8)	52 (24.4)	19 (12.6)	34 (24.3)	39 (17.1)
Police pressure	8	12 (3.3)	5 (2.4)	7 (4.6)	7 (5.0)	5 (2.2)
**Working on Sundays**	6	164 (44.4)	77 (36.2)	84 (55.3)	62 (44.3)	102 (44.5)
**Workdays (per week)**	5					
≤ 5 days		26 (7.0)	20 (9.3)	6 (3.9)	12 (8.6)	14 (6.1)
6 days		194 (52.4)	127 (59.4)	65 (42.8)	71 (50.7)	123 (53.5)
7 days		150 (40.5)	67 (31.3)	81 (53.3)	57 (40.7)	93 (40.4)

97% of the children and adolescents reported having suffered occupational injuries during their lifetime. The lifetime prevalence of mild injuries was 72%, with cuts and abrasions being the most frequent injuries (Table 3). 3% of the study population reported having ever suffered severe injuries (amputations, luxation and fractures). Most frequent causes of injuries were falls (11%) and car accidents (9%); 3% reported to suffer from physical violence at the workplace.

**Table 3:** **Lifetime prevalence of occupational injuries, total and stratified for gender and age**

Category	n _missing_	n (%)	n (%)	n (%)	n (%)	n (%)
			male	female	9–14 years	15–17 years
**None**	10	**11 (3.0)**	**5 (2.4)**	**5 (3.3)**	**7 (5.1)**	**4 (1.8)**
**Any mild injuries**	10	**263 (72.1)**	**147 (69.7)**	**114 (76.5)**	**105 (76.1)**	**157 (69.5)**
Of these:						
Cuts	1	207 (55.3)	105 (48.8)	99 (63.9)	74 (52.1)	133 (57.3)
Abrasions	0	201 (53.6)	125 (57.9)	74 (47.7)	68 (47.6)	133 (57.3)
Pricks	10	1 (0.3)	1 (0.5)	0 (0.0)	0 (0.0)	1 (0.4)
Mild burns	1	64 (17.1)	31 (14.4)	33 (21.3)	27 (18.9)	37 (16.0)
**Injuries caused by falls**	11	**40 (11.0)**	**24 (11.4)**	**16 (10.7)**	**12 (8.7)**	**28 (12.4)**
**Injuries caused by car accidents**	0	**33 (8.8)**	**19 (9.0)**	**12 (8.1)**	**8 (5.8)**	**25 (11.0)**
**Injuries caused by physical violence**	11	**10 (2.7)**	**8 (3.8)**	**2 (1.3)**	**2 (1.4)**	**8 (3.5)**
**Any severe injuries**	10	11 (3.0)	**10 (4.7)**	**1 (0.7)**	**5 (3.6)**	**6 (2.6)**
Of these:						
Fractures	11	5 (1.4)	5 (2.4)	0 (0.)	1 (0.7)	4 (1.8)
Luxations	11	1 (0.3)	1 (0.5)	0 (0.)	0 (0.)	1 (0.4)
Amputations	0	5 (1.3)	4 (1.9)	1 (0.6)	4 (2.8)	1 (0.4)

### Potential risk factors for occupational injuries

According to the bivariate analyses, higher income was statistically significantly associated with higher lifetime prevalence of falls, severe injuries and injuries due to violence (Table 4). The lifetime prevalence of injuries caused by violence was also elevated in the construction sector (20%,  = 0.008). The lifetime prevalence of car accidents was borderline significantly higher in the service sector (14%) and in construction (14%;  = 0.10). In addition, age, being born in Cusco and lower number of working days per week were associated with a higher lifetime prevalence of car accidents. Boys (6.2%) were more prone to severe injuries than girls (0.8%;  = 0.03).

**Table 4:** **Prevalence of different types of occupational injuries by potential risk factors**

	N _missing_	Prevalence of falls ^1^		Prevalence of injuries caused by car accidents ^2^		Prevalence of injuries caused by physical violence at work ^3^		Prevalence of severe injuries ^4^	
N		313		306		283		284	
Overall n (%)		40 (12.8%)		33 (10.8)		10 (3.5)		11 (3.9)	
		n (%)	p_Chi_^2^_exact_	n (%)	p_Chi_^2^_exact_	n (%)	p_Chi_^2^_exact_	n (%)	p_Chi_^2^_exact_
Age	0		0.226		0.088		0.212		1.00
9–14 years		12 (9.7)		8 (6.7)		2 (1.7)		5 (4.3)	
15–17 years		28 (14.8)		25 (13.4)		8 (5.0)		6 (3.6)	
Gender	4		0.733		0.703		0.196		0.027
Male		24 (13.6)		19 (11.1)		8 (5.0)		10 (6.2)	
Female		16 (11.9)		12 (9.2)		2 (1.7)		1 (0.8)	
Born and raised in Cusco	6		0.364		0.001		1.00		1.00
No		33 (11.8)		23 (8.5)		9 (3.5)		10 (3.9)	
Yes		5 (17.9)		10 (30.3)		1 (4.2)		1 (4.2)	
Living alone	9		0.544		0.831		0.706		0.470
No		27 (11.7)		26 (11.3)		7 (3.3)		7 (3.3)	
Yes		11 (14.9)		7 (10.0)		3 (4.5)		4 (6.0)	
Education	5		0.191		0.329		1.00		0.358
Primary		8 (8.3)		14 (13.7)		3 (3.3)		2 (2.2)	
Secondary		30 (14.1)		19 (9.4)		7 (3.7)		9 (4.7)	
Healthcare coverage	8		0.333		0.488		0.620		1.00
No		37 (13.0)		31 (11.1)		10 (3.9)		10 (3.9)	
Yes		1 (4.5)		1 (4.5)		0 (0.0)		1 (4.5)	
Sector	1		0.309		0.097		0.008		0.164
Retail		13 (15.5)		3 (4.1)		2 (2.7)		0 (0.0)	
Manufacturing		3 (9.4)		2 (6.5)		2 (6.5)		2 (6.5)	
Service		20 (11.0)		26 (13.9)		3 (1.8)		9 (5.3)	
Construction		4 (25.0)		2 (14.3)		3 (20.0)		0 (0.0)	
Working on Sundays	6		1.00		0.463		0.352		0.760
No		21 (12.2)		16 (9.6)		4 (2.6)		7 (4.4)	
Yes		17 (12.4)		17 (12.4)		6 (4.8)		4 (3.2)	
Working days (per week)	5		0.850		0.006		0.493		0.703
≤5 days		3 (16.7)		7 (31.8)		0 (0.0)		0 (0.0)	
6 days		20 (12.3)		16 (10.1)		4 (2.7)		7 (4.7)	
7 days		15 (11.6)		10 (8.1)		6 (5.0)		4 (3.4)	
Income (per day)	27		0.047		0.127		<0.001		0.034
0–9 PEN		13 (9.8)		9 (7.0)		1 (0.8)		4 (3.3)	
10–19 PEN		12 (11.3)		17 (15.3)		1 (1.1)		2 (2.1)	
≥20 PEN		12 (23.1)		5 (11.1)		7 (14.9)		5 (11.1)	

p_Chi_^2^_exact_: exact p-Chi^2^ value

N_missing_: number of missing observations per item (item non-response).

^1^excluding those who suffered injuries caused by car accidents, physical violence or luxation, fractures or amputations.

^2^excluding those who suffered injuries caused by falls, physical violence or luxation, fractures or amputations.

^3^excluding those who suffered injuries caused by falls, car accidents or luxation, fractures or amputations.

^4^luxation, fractures, amputations; excluding those who suffered injuries caused by falls, car accidents, or physical violence.

In the multiple logistic regression models, higher income was confirmed as statistically significant predictor of falls (OR 2.75; 95% CI 1.16-6.51) and injuries caused by violence (12.10; 1.26-116.01). Being born in Cusco (3.64; 1.44-9.26), less working days (0.24; 0.07-0.74) and working in the service sector (4.24; 1.17-15.39) were statistically significantly associated with injuries caused by car accidents. For severe injuries, no risk factor reached the level of statistical significance after mutual adjustment for the other variables in the model (Table 5).

**Table 5:** **Results of the multiple logistic regression models analyzing the association between potential predictors and occupational injuries by type of injuries**

	Falls ^1^	Injuries caused by car accidents ^2^	Injuries caused by physical violence at work ^3^	Severe injuries (Luxation, fractures, amputations) ^4^
N	290	303	262	262
Age	-		-	-
9–14 years		1		
15–17 years		2.12 (0.88–5.13)		
Gender	-	-	-	
Female				1
Male				6.38 (0.76–53.90)
Born and raised in Cusco	-		-	-
No		1		
Yes		3.64 (1.44–9.26)		
Working days (per week)	-		-	-
≤5 days		1		
6 days		0.26 (0.08–0.84)		
7 days		0.24 (0.07–0.74)		
Sector	-			-
Retail		1	1	
Manufacturing		2.00 (0.30–13.50)	1.42 (0.17–11.66)	
Service		4.24 (1.17–15.39)	0.60 (0.08–4.61)	
Construction		5.33 (0.72–39.17)	3.39 (0.45–25.64)	
Income (per day)		-		
0–9 PEN	1		1	1
10–19 PEN	1.17 (0.51–2.68)		1.07 (0.06–17.60)	0.49 (0.08–2.66)
≥20 PEN	2.75 (1.16–6.51)		12.10 (1.26–116.01)	2.15 (0.52–8.28)

Odds Ratios with 95% Confidence Interval mutually adjusted for all other variables in each model.

^1^excluding those who suffered injuries caused by car accidents, physical violence or luxation, fractures or amputations.

^2^excluding those who suffered injuries caused by falls, physical violence or luxation, fractures or amputations.

^3^excluding those who suffered injuries caused by falls, car accidents or luxation, fractures or amputations.

^4^excluding those who suffered injuries caused by falls, car accidents, or physical violence.

## Discussion

This study examined the lifetime prevalence of and risk factors for occupational injuries among working children and adolescents (10–17 years old) who attend public night schools in Cusco Province. Three children of the study population were as young as 10 years, six only 11 years old. About one quarter of the kids were living alone, many of them not coming from Cusco but moved there looking for better opportunities or because of poverty. Average income was well below the minimum wage in Perú. While the majority of occupational injuries reported might be considered mild, 3% of the study population reported having ever suffered severe injuries. Furthermore, 3% suffered from physical violence at work. According to our results, children working in the service sector are at increased risk of traffic injuries. Interestingly, the main predictor of falls and violence at work was higher income.

One limitation of our study might be that we considered lifetime prevalence of occupational injuries instead of the annual incidence [9–11]. The reason for that was the use of a questionnaire instrument that was employed earlier in other Latin American cities [6]. Within this survey the lifetime prevalence of occupational injuries in Latin American child street laborers from Bogotá, Sao Paulo, Quito and Lima was much lower (40%) than in our study (92%). One reason for this huge difference might be the different approaches to contact children: while our children were invited to participate in an environment of trust (= the school) participants in the earlier study were approached on the street. As a result, our study reached a very high response thus reducing the potential of selection bias. Differences in reporting might be an issue and could have resulted in over-reporting in our and under-reporting in the previous study [6]. However, no data on reliability or validity of the questionnaire instrument are available. Furthermore, our study included older children only (10–17 years vs. 5–17 years in [6]) and increasing age might have resulted in a higher lifetime prevalence of injury in our study population.

One limitation of assessing the lifetime prevalence of occupational injuries is that the reported injury could have potentially occurred in a job other than the current (reported) one. In addition, recall bias might result in an underestimation of the true prevalence. Adaptations of the questionnaire need to be considered in order to gain information on the time and the circumstances of the injury events, thereby making sure to account for the occupation in which the related injury occurs. Also, the questionnaire did not solicit detailed information about injuries caused by falls, car accidents and physical violence. Specification of the outcome and severity of these injuries should be added as further items to the questionnaire. However, one has to carefully consider the length of the questionnaire instrument which especially in this target group of working children with a broad age range has to be kept short. As any cross-sectional design, our study does not allow any inference of causal directions.

Another drawback is the limited power of our study which occurred despite having a large study population and an unfortunately high lifetime prevalence of occupational injuries. As a result, we were only able to describe the problem and identify the most important risk factors for the injuries under study. Risk factors having a smaller effect on occupational injuries could not be identified. However, the number of participants was limited by design - as no more night schools in Cusco Province exist we unfortunately could not invite more children using this access way which we considered the best approach to obtain a high response and comparable study population across the province.

Due to the differing abilities to read of the study population the data collection was carried out by interview. In order to minimize interviewer bias the interviewer read the questions without any further explanation. Furthermore, all interviews were conducted by the same person. Finally, potential confounding was taken into account in the analyses step.

Children working in the service sector had a more than four times higher risk of injuries caused by car accidents than children working in the retail sector. Many of these service activities take place on the street, such as washing and guarding cars, serving as bus attendants, polishing shoes, transporting goods or collecting recyclables [4]. Child street workers in such jobs are prone to a constant risk of traffic accidents. Although it should be a long-term goal to reduce the overall number of children and adolescents working on the street, the implementation of safety equipment, e.g. reflective vests for bus attendants, might decrease the risk of traffic accidents. Moreover, within the population of working students, the schools could lead the way to design intervention strategies that prevent children from occupational injuries, for example by increasing the children’s knowledge of occupational hazards and by providing safety training as well as skill building programs [6, 12].

Children who were born and raised in Cusco province had almost a four times higher risk of injuries caused by car accidents than children who had moved to Cusco. The reason for the high risk car accidents in children who were born and raised in Cusco is probably due to the longer duration of work on the street.

Children earning 20 or more PEN/day were at risk of injuries caused by falls and injuries caused by physical violence at work. Factors associated with higher income were older age (85% of those with high income being 15–17 years old as compared to 56% in the rest of the population, p_Chi_^2^ < 0.001), 85% of them being male (vs. 53%; p_Chi_^2^ < 0.001). Almost 50% of them reported to live alone (46% vs. 19%; p_Chi_^2^ < 0.001). About 40% of them were working in construction (18% vs. 3%) or carpentering (18% vs. 9%; p_Chi_^2^ < 0.001; data not shown). So this group of mainly older boys mostly living alone – without family protection - and working in typically “male” jobs seems to be an unprotected high risk group which needs special attention and should be addressed in future studies.

As this study focuses on a subgroup of child laborers, the findings apply to working children and adolescents attending night schools and living in a region characterized by continuous urbanization and tourism. Those with worse working conditions, more demanding jobs as well as those who have suffered more severe injuries might be especially underrepresented in our study population. We also lack information about the employment conditions of our study population. Therefore, results can neither be generalized to all working children in Cusco nor to the whole of Perú or other Latin American countries.

## Conclusions

A large number of working children and adolescents in Cusco Province who combine school and work experience work-related accidents and many of them even suffer from severe occupational injuries. Higher income seems to be related to a higher life-time prevalence of injury. Policies against child labor should particularly address street child labor within the service sector as well as older boys living outside the family who might accept bad working conditions for a higher income.
